# Supplementary material for: Unravelling sex differences in the genetic architecture of anxiety
Source: Psychol Med. 2026 Jun 11;56:e191. doi: 10.1017/S0033291726104760 (PMC13280693; doi:10.1017/S0033291726104760)

Supplementary Information For

**Unravelling Sex Differences in the Genetic Architecture of Anxiety**

Jihua Hu^1,2^, Michelle K. Lupton^1,2,3^, Enda M. Byrne^4^, Nicholas G. Martin^1^, David C. Whiteman^2,5^, Catherine M. Olsen^2,5^, Jodi T. Thomas^1,2^, Sarah E. Medland^1,6,7^, Katrina L. Grasby^1,2,3#^, Brittany L. Mitchell^1,2,3#^

**Contents**

[Phenotype definition 2](#_Toc226472990)

[Phenotype in the UK Biobank 2](#_Toc226472991)

[Phenotype in the All of Us 3](#_Toc226472992)

[GWAS of qualitative GAD-7 in the UK Biobank 4](#_Toc226472993)

[GWAS of EHR-based anxiety cases in AoU females 5](#_Toc226472994)

[Polygenic scores 5](#_Toc226472995)

[References 7](#_Toc226472996)

[Supplementary Figures 8](#_Toc226472997)

[Figure S1: Manhattan plot of sex-combined and sex-specific GWAS in the UK Biobank 8](#_Toc226472998)

Figure S2: Manhattan plot of sex-combined and sex-specific GWAS in the All of Us…………………………….9

[Figure S3: Sex-specific polygenic risk scores (PRS) in the clinical sample. 10](#_Toc226472999)

**Supplementary**

Phenotype definition

In this study, we identified participants who met DSM-5 criteria for the following anxiety disorders (1): agoraphobia, social phobias, specific phobias, generalized anxiety disorders, panic disorders and other phobic anxiety disorders.

Phenotype in the UK Biobank

Lifetime anxiety cases in the UKB were defined as individuals that met any of the four following criteria:

i) Individuals who reported having been diagnosed with at least one anxiety disorder by a professional using ICD10 criteria;

ii)  Individuals diagnosed with an anxiety disorder by a professional;

iii) Individuals who met the anxiety criteria of the Composite International Diagnostic Interview (CIDI) short-form questionnaire (2);

iv) Individuals whose total sum score was above or equal to eight in the 7-item general anxiety disorder (GAD-7) scale (3).

We chose eight as the cut-off for the GAD-7 as this has been shown to give the best performance in terms of sensitivity and specificity for classifying anxiety disorders (4). For participants who answered the GAD-7 on multiple occasions, we used the highest sum score as their final score. Among the 15,910 participants with a GAD-7 score ≥ 8, 65.43% (n = 10,411) also met criteria for anxiety based on at least one of the other definitions. The detailed questions and related data fields in the UK Biobank are listed in Table S1.

Participants were classified as controls if they answered no to the following questions *“In your life, have you suffered from a period of mental distress that prevented you from doing your usual activities?”* and *“In your life, did you seek or receive help from a professional (medical doctor, psychologist, social worker, counselor, nurse, clergy, or other helping professional) for mental distress, psychological problems or unusual experiences?”*. In addition, we screened out participants who had been diagnosed with any mental health conditions by a professional or with an ICD10 code, or with a GAD-7 sum score greater than seven.

| To document the degree of overlap across anxiety phenotypes in the UK Biobank, we provide a pairwise case counts for all combinations of definitions. | **GAD7 2017** | **GAD7 2023** | **MHC 2023** | **MHC 2017** | **CIDI-SF** | **Panic Disorder** | **ICD 10** |
| --- | --- | --- | --- | --- | --- | --- | --- |
| **GAD7 2017** | 10,354 | 2,635 | 3,330 | 4,710 | 3,344 | 1,219 | 1,413 |
| **GAD7 2023** | 2,635 | 10,770 | 5,641 | 2,918 | 1,884 | 2,293 | 1,430 |
| **MHC 2023** | 3,330 | 5,641 | 30,403 | 12,498 | 4,570 | 6,138 | 4,273 |
| **MHC 2017** | 4,710 | 2,918 | 12,498 | 27,613 | 6,184 | 3,611 | 3,689 |
| **CIDI-SF** | 3,344 | 1,884 | 4,570 | 6,184 | 11,039 | 1,807 | 1,593 |
| **Panic Disorder** | 1,219 | 2,293 | 6,138 | 3,611 | 1,807 | 9,433 | 1,217 |
| **ICD 10** | 1,413 | 1,430 | 4,273 | 3,689 | 1,593 | 1,217 | 27,474 |

Phenotype in the All of Us

Lifetime anxiety cases in the AoU dataset were defined as individuals who either had a documented diagnosis in their electronic health record (EHR) of generalized anxiety disorder, panic disorder, or phobic disorder, or who selected “self” in response to the survey question: “Including yourself, who in your family has had anxiety reaction/panic disorder?”

Among females, 17,059 were identified from EHR records and 27,963 from self-report, including 7,034 who met both criteria. Among males, 6,846 were identified from EHR records and 8,057 from self-report, including 1,990 who met both criteria.

Controls were defined as participants who had both EHR and genotyping data available, but did not have any documented EHR diagnosis corresponding to the following conditions:  Depressive disorder (ID: 440383), Anxiety (ID: 441542), Bipolar disorder (ID: 436665), Schizophrenia (ID: 435783), or Mood disorder (ID: 444100).

GWAS of qualitative GAD-7 in the UK Biobank

We conducted a separate GWAS of the GAD-7 quantitative score in order to evaluate the consistency of this measure with our binary lifetime anxiety phenotype. Among 191,028 participants who completed all seven items, we excluded those with a score of 0 (leaving 106,944) due to the zero-inflated distribution and to focus on individuals with at least minimal anxiety symptoms. Scores were rank-based inverse-normal transformed prior to association testing in REGENIE (v2.2.4) (5).

GWAS was also performed for the other binary definitions of anxiety. Genetic correlation analysis using LD Score Regression (LDSC) (6) demonstrated a high correlation between the quantitative GAD-7 phenotype and the binary lifetime anxiety definition  (rg=0.84 (0.04), P=4.59^-118^).

**GWAS of GAD7 binary phenotype in the UK Biobank**

Because the GAD-7 is a dimensional measure of anxiety symptoms rather than a diagnostic instrument, we conducted a sensitivity analysis to determine whether using the minimal clinical cutoff (score ≥8) yields a case definition that is genetically comparable to our disorder-level anxiety phenotypes. We constructed a binary GAD-7 phenotype in UK Biobank by defining cases as participants with GAD-7 ≥8, using the same set of controls as in the primary analyses.

In parallel, we performed a second binary GWAS using an independent anxiety case definition based on self-reported diagnoses, CIDI-SF assessments, panic disorder items, and ICD-10 codes; importantly, individuals identified only through the GAD-7 cutoff were excluded from this phenotype. The same control group was used.

We then estimated the genetic correlation between the two binary GWASs. The resulting estimate, rg = 0.89 (SE = 0.026), P = 8.71 × 10⁻²⁵⁶, indicates extremely strong shared genetic liability between the GAD-7 cutoff phenotype and established diagnostic-level anxiety case definitions.

GWAS of EHR-based anxiety cases in AoU females

In the AoU dataset, more anxiety cases were identified from self-reports than from EHR records. To assess whether including self-reported cases biased the results, we performed a GWAS restricted to female participants with EHR-based diagnoses using REGENIE. We then estimated the genetic correlation between this EHR-only GWAS and our finalized female GWAS in AoU, which included both EHR- and self-reported cases. The correlation was very high (rg=0.95 (0.03), P=8.65^-177^), supporting the validity of including self-reported cases in the final analysis.

Polygenic scores

To test whether our results were able to significantly predict anxiety in an independent sample, we calculated polygenic scores (PGS) using SBayesR (7). SBayesR is a Bayesian technique that estimates the effect of SNPs from multi-normal distributions which may reflect the true distribution of genetic variants. The estimated PGS were standardized using the *scale()* function in R (v4.2.0).

We tested the predictive ability of PGS derived from our GWAS results in the QSkin Sun and Health Study (QSkin). QSkin is a population-based cohort focused on skin cancer and melanoma in Queensland, Australia (8). Participants in QSkin completed online questionnaires, and 2,341 individuals who responded 'yes' to the question 'Have you ever been diagnosed with, experienced or been treated for anxiety?' were classified as cases, and 12,722 individuals with no history of psychiatric disorders were used as controls.

To control for potential familial relationships in the QSkin dataset, we conducted a restricted maximum likelihood (REML) analysis using Genome-wide Complex Trait Analysis (GCTA) (v 1.91.7). We estimated the variance in lifetime anxiety status explained by sex-specific GWAS-derived PGS (PGS_F_ for females and PGS_M_ for males) separately in 8,009 females (N cases = 1,528) and 7,057 males (N cases = 819). We adjusted the model for the first 10 PCs. Similarly, we estimated PGS_C_ from the combined GWAS that included females and males to predict anxiety in all 15,066 individuals, with the model further adjusted for sex as a covariate.

To examine whether our PGS results were influenced by differences in sample sizes, and thus statistical power, across our discovery GWASs, we downsampled the number of cases and controls in the female-specific GWAS to match those in the male-specific GWAS in both UKB and AoU. These downsampled female GWAS results were then meta-analysed and used to generate PRS. To ensure comparable evaluation of PGS predictions, we also downsampled the female prediction cohorts to the same sample size as males (QSkin, AGDS/QSkin, PISA, and AGDS). Downsampling was performed through random selection using the *sample()* function in R(v4.2.0). We then repeated the regression analyses using these downsampled results, following the same procedure as above.

Additionally, we tested the association between PGS_C_, PGS_F_ and PGS_M_ with a continuous measure of current anxiety symptoms, as measured by the GAD-7. The GAD-7 data were available for 4,868 individuals (3,263 females and 1,605 males) in the Prospective Imaging Study of Ageing: Genes, Brain and Behaviour (PISA) (9). PISA is an Australian-based cohort of middle-aged and older participants investigating risk factors and biomarkers for dementia. Participants completed an online survey which included the GAD-7. Associations of PGS and GAD-7 were tested using the REML approach in GCTA, similar to the analysis of anxiety in QSkin.

Finally, we tested the PGS predictions of both lifetime anxiety and current anxiety (GAD-7) in a secondary sample, the Australian Genetics of Depression Study (AGDS). AGDS is an Australia-based cohort, including approximately 17,000 genotyped participants (10) that report a history of depression. Participants in AGDS completed online questionnaires, and those who reported a diagnosis of generalized anxiety disorders were classified as lifetime anxiety cases, and the individuals reported no history of psychiatric disorders from QSkin were used as the controls. There are 14,759 females (cases=8,278) and 9,140 males (cases=2,902) for prediction of lifetime anxiety. GAD-7 data were available for 7,282 individuals (5,556 females and 1,681 males) in AGDS. While larger in size, it is important to note that in AGDS all the anxiety cases have comorbid depression. Therefore, this sensitivity analysis may distinguish prediction between population-based, non-comorbid cohorts and clinical, comorbid-depression cohorts. All models were adjusted for the same covariates as in the approach above and GCTA was used for analyses.

References

1. Diagnostic and statistical manual of mental disorders: DSM-5^TM^, 5th ed. Arlington, VA, US: American Psychiatric Publishing, Inc.; 2013. xliv, 947 p. (Diagnostic and statistical manual of mental disorders: DSM-5^TM^, 5th ed).

2. Kessler RC, Andrews G, Mroczek D, Ustun B, Wittchen HU. The World Health Organization Composite International Diagnostic Interview short-form (CIDI-SF). Int J Methods Psychiatr Res. 1998;7(4):171–85.

3. Spitzer RL, Kroenke K, Williams JBW, Löwe B. A Brief Measure for Assessing Generalized Anxiety Disorder: The GAD-7. Arch Intern Med. 2006 May 22;166(10):1092–7.

4. Johnson SU, Ulvenes PG, Øktedalen T, Hoffart A. Psychometric Properties of the General Anxiety Disorder 7-Item (GAD-7) Scale in a Heterogeneous Psychiatric Sample. Front Psychol. 2019 Aug 6;10:1713.

5. Mbatchou J, Barnard L, Backman J, Marcketta A, Kosmicki JA, Ziyatdinov A, et al. Computationally efficient whole-genome regression for quantitative and binary traits. Nat Genet. 2021 July;53(7):1097–103.

6. Bulik-Sullivan BK, Loh PR, Finucane HK, Ripke S, Yang J, Patterson N, et al. LD Score regression distinguishes confounding from polygenicity in genome-wide association studies. Nat Genet. 2015 Mar;47(3):291–5.

7. Lloyd-Jones LR, Zeng J, Sidorenko J, Yengo L, Moser G, Kemper KE, et al. Improved polygenic prediction by Bayesian multiple regression on summary statistics. Nat Commun. 2019 Nov 8;10(1):5086.

8. Olsen CM, Green AC, Neale RE, Webb PM, Cicero RA, Jackman LM, et al. Cohort profile: the QSkin Sun and Health Study. Int J Epidemiol. 2012 Aug;41(4):929–929i.

9. Lupton MK, Robinson GA, Adam RJ, Rose S, Byrne GJ, Salvado O, et al. A prospective cohort study of prodromal Alzheimer’s disease: Prospective Imaging Study of Ageing: Genes, Brain and Behaviour (PISA). NeuroImage Clin. 2021 Jan 1;29:102527.

10. Byrne EM, Kirk KM, Medland SE, McGrath JJ, Colodro-Conde L, Parker R, et al. Cohort profile: the Australian genetics of depression study. BMJ Open. 2020 May 1;10(5):e032580.

Supplementary Figures

Figure S1: Manhattan plot of sex-combined and sex-specific GWAS in the UK Biobank


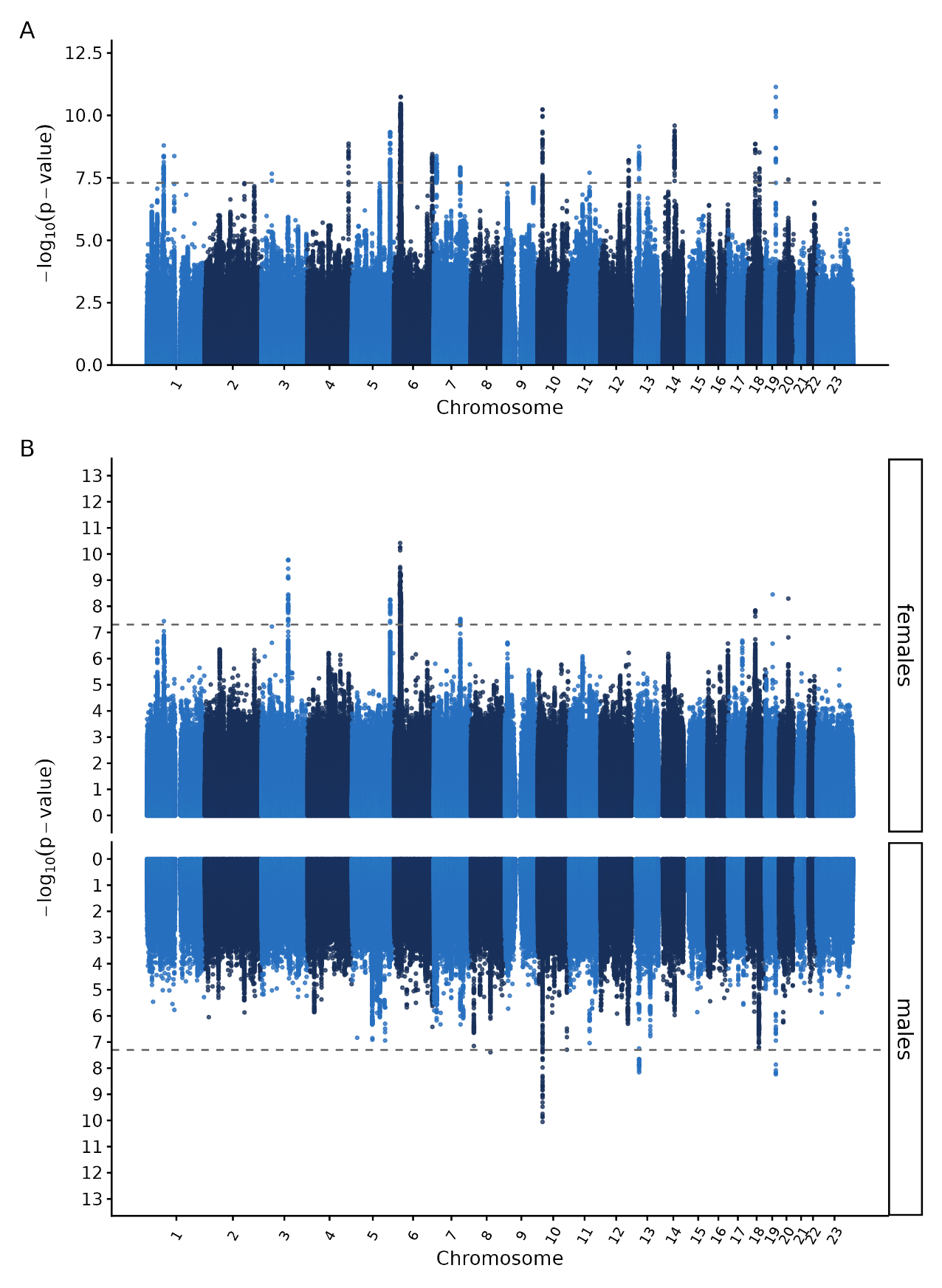


Figure S2:  Manhattan plot of sex-combined and sex-specific GWAS in the All of Us

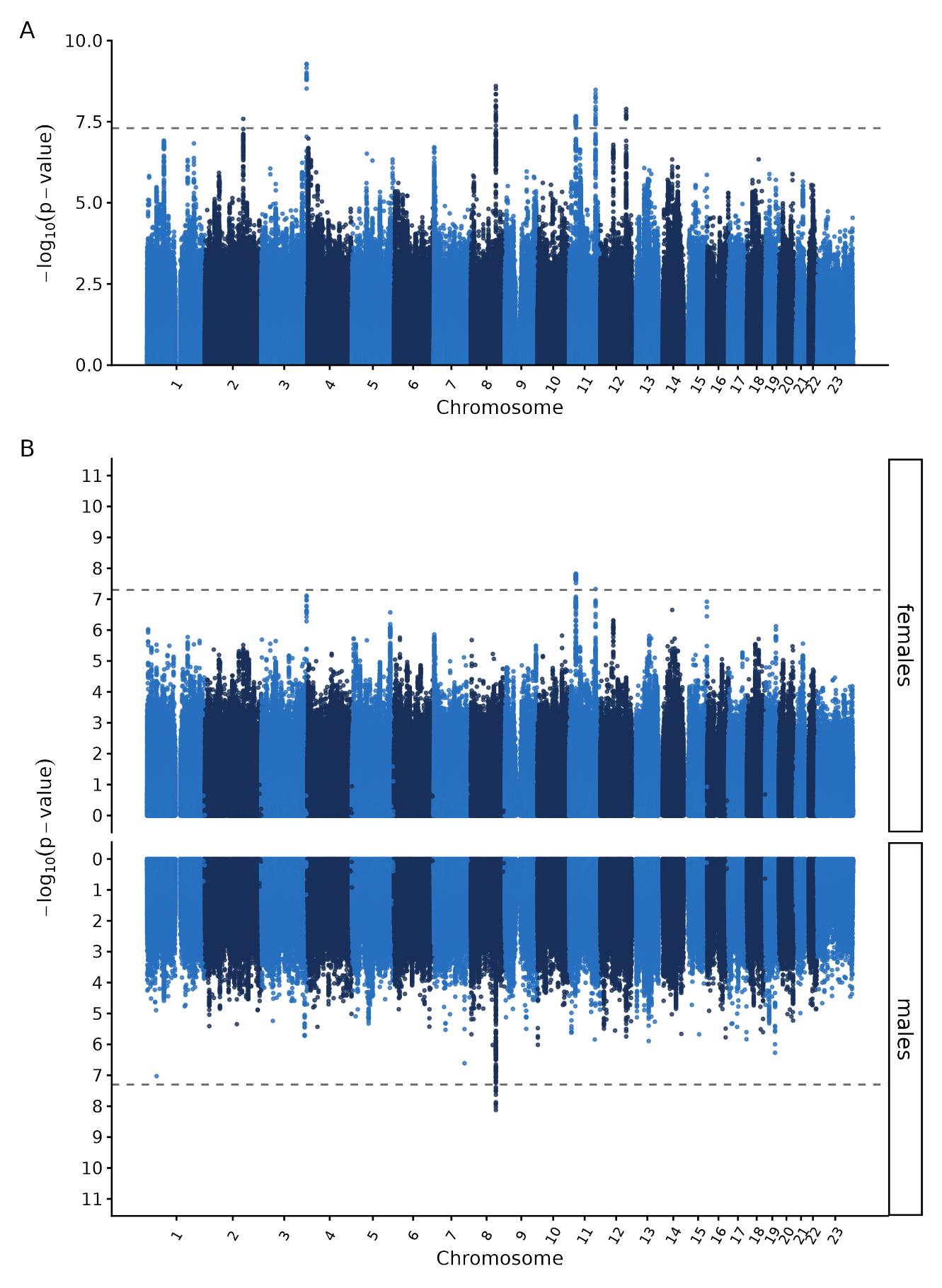


Figure S3: Sex-specific polygenic risk scores (PRS) in the clinical sample.

**3A**: PGS prediction for lifetime anxiety in AGDS/Qskin. **3B**: PGS prediction of GAD-7 in AGDS.


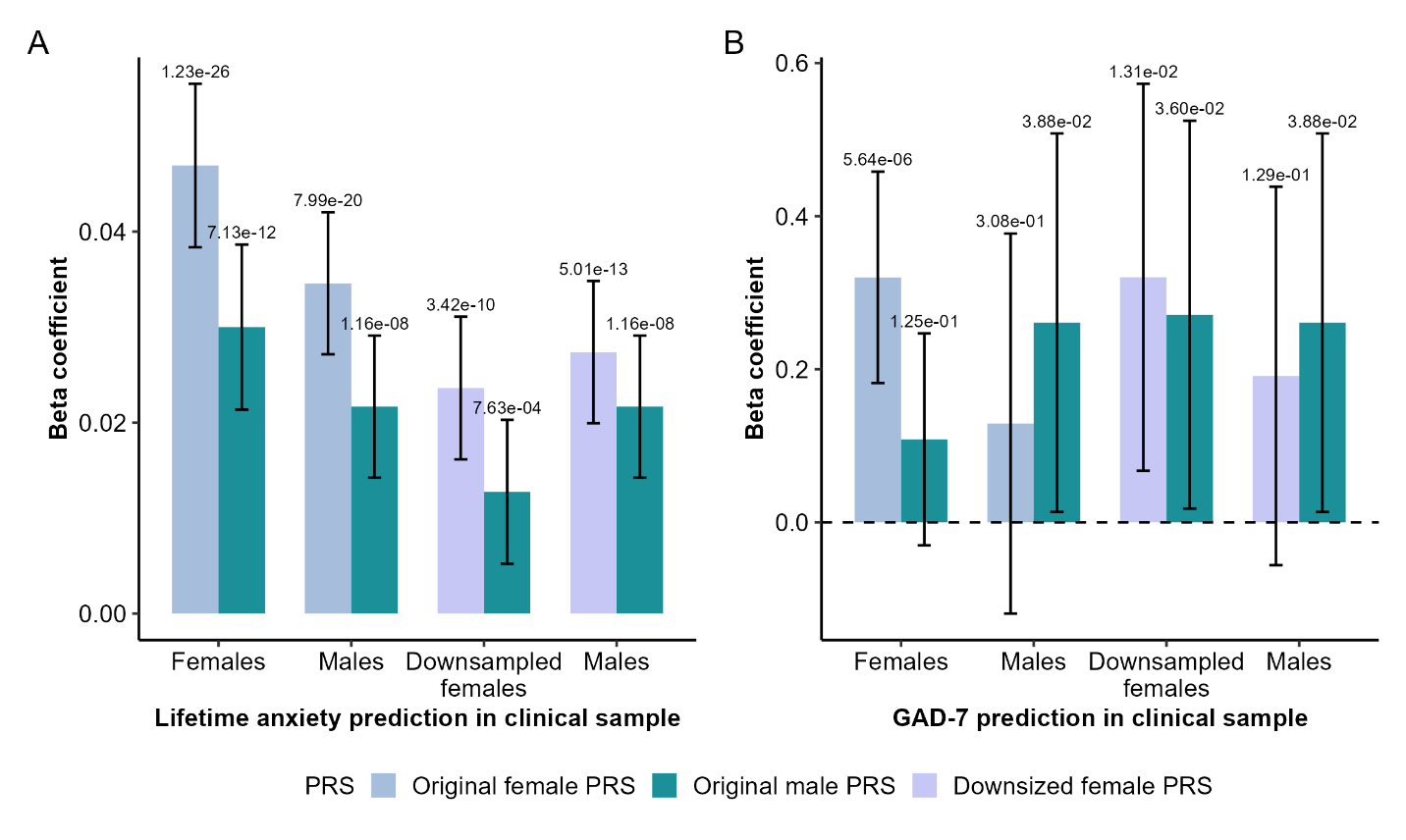

Supplement: Hu et al. supplementary material 1 — Hu et al. supplementary material [file S0033291726104760sup001.docx]
